# Prevalence of Obesity and Its Association with Socioeconomic Factors in Elderly Iranians from Razavi-Khorasan Province

**DOI:** 10.1100/tsw.2009.139

**Published:** 2009-11-18

**Authors:** Mohsen Nematy, Ali Sakhdari, Parnian Ahmadi-Moghaddam, Maliheh Aliabadi, M. Kimiagar, A. A. Ilaty, M. Azimi-Nezhad, M. T. Shakeri, M. Ghayour-Mobarhan, A. Sahebkar, G. Ferns

**Affiliations:** ^1^ Department of Nutrition and Biochemistry, Faculty of Medicine, Mashad University of Medical Sciences (MUMS), Mashhad, Islamic Republic of Iran; ^2^ Cardiovascular Research Center, Avicenna Research Institute, MUMS, Mashhad, Islamic Republic of Iran; ^3^ Razavi-Khorasan Health Center, MUMS, Mashhad, Islamic Republic of Iran; ^4^ Department of Human Nutrition, Beheshti University of Medical Sciences, Tehran, Islamic Republic of Iran; ^5^ Community Health and Statistics Department, Faculty of Medicine, MUMS, Mashhad, Islamic Republic of Iran; ^6^ Centre for Clinical Science and Measurement, University of Surrey, Guildford, Surrey, UK

**Keywords:** body mass index (BMI), obesity, free-living elderly people (community dwellers), malnutrition, socioeconomic status

## Abstract

There are few data regarding the prevalence of obesity and its socioeconomic determinants among elderly individuals, particularly in Iran. We wished to determine the prevalence of overweight and obesity in free-living elderly people and the relationship to nutritional and socioeconomic factors in the Razavi-Khorasan province of Iran. Free-living elderly persons (917 males/1045 females), aged ?60 years, were recruited using cluster sampling. Overweight and obesity were evaluated using body mass index (BMI) and subjects were categorized as thin (BMI <18.5 kg/m^2^), normal (18.5–24.9 kg/m^2^), overweight (25–29.9 kg/m^2^), and obese (?30 kg/m^2^). The association between the prevalence of overweight or obesity with socioeconomic and demographic factors, including gender, place of residence, literacy, type of living, source of income, use of supplements during the past 3 months, and employment status, was examined using regression analysis. The distribution of BMI values indicated that 13, 46.5, 28.9, and 11.7% of the total population were thin, normal, overweight, and obese, respectively. The prevalence of central obesity was higher among Iranian women than men (63.1 vs. 18.6%, respectively). Regression analysis results indicated that gender (*p* < 0.001), place of residence (*p* < 0.001), literacy (*p* = 0.01), and source of income (*p* < 0.001) were significantly associated with the incidence of overweight or obesity. This study showed that 40.6% of elderly subjects were overweight or obese. Results reinforce the need to plan strategies for primary prevention of this fast-growing public health problem.

## INTRODUCTION

Obesity is becoming a major public health problem, being a predisposing factor for diabetes mellitus, cardiovascular disease, osteoarthritis, and sleep apnea[1]. The recent rise in the prevalence of obesity has been attributed to environmental and societal change, rather than individual behavior[2]. In particular, there has been a general trend to decreased physical activity and increased dietary calorie intake[3].

Obesity is highly prevalent among elderly people[4]. As the elderly are a particularly vulnerable group, obesity and its complications may lead to disability and so it is a major health concern in this age group[3,5,6]. A recent study shows age-related changes with increases in body fat composition due to increasing age[7]. In a study conducted in Spain, the prevalence of overweight and obesity was reported to be 49 and 31.5%, respectively, in men, and also 39.8 and 40.8%, respectively, in women[8]. It was also found that 34% of men and 40.3% of women were obese in northern India[9]. The rate of obesity among American elderly was reported to be 33.4 and 30.2% in females and males, respectively[10]. On the other hand, the prevalence of BMI ≥25 among elderly males and females from the U.K. was reported to be 60 and 60.2%, respectively[11]. In Saudi Arabia, the prevalence of central obesity in the general population, based on waist circumference and the ratio of waist circumference to hip circumference, was found to be 34.4 and 43.5%, respectively[12]. Among elderly women, obesity has been reported to be positively associated with a fasting blood sugar ≥126 mg/dl[13]. Moreover, obesity is associated with increased mortality in elderly individuals that may be associated with the presence of cardiovascular risk factors, such as a higher level of blood lipids[14]. Socioeconomic studies in a Korean population have shown that individuals with high or moderate income were more prone to overnutrition and obesity[15]. However, this finding was not confirmed by some other surveys performed in the populations of the U.S. and U.K. for whom lower socioeconomic status was associated with an increased prevalence of obesity[16,17,18,19]. To our knowledge, the prevalence of overweight and obesity are unknown in elderly Iranians. Therefore, this study may help health care professionals to understand which socioeconomic factors are associated with overweight and obesity.

## METHODS

Data for the present study were obtained from subjects recruited into an earlier study that investigated the prevalence of malnutrition among the elderly[20]. In this previous study, subjects (n = 2000) were recruited using a cluster-stratified sampling method from the free-living elderly people of Razavi-Khorasan province, from a total elderly population of about 460,000, and included individuals from rural and urban areas over 60 years of age. Razavi-Khorasan province had three clusters for every urban and rural district, which were selected randomly. Subjects were selected using the probabilities proportional to their district population in order to produce the target sample size. Among the selected subjects, 1962 completed the nutritional status assessment. To assess socioeconomic indices, questions were set as part of the questionnaire. Information from interview, physical examination, and data collection forms of these 1962 subjects were used for the current study.

In the aforementioned study, weight of subjects was assessed using a digital portable scale, measuring to the nearest 0.1 kg. Height was measured to the nearest 0.5 cm in those who were able to stand upright, using a stadiometer. In those who were not able to stand, height was measured using the demispan method (sternal notch to finger roots with arm outstretched laterally)[21]. Body mass index (BMI) and waist circumference were used to diagnose overweight and obesity. BMI (the most commonly used index of weight and height) is calculated using the following equation: Weight (kg)/Height ($m^2$)[22]. A BMI of <19 kg/$m^2$ was considered thin, 19–24.9 kg/$m^2$ normal, 25–29.9 kg/$m^2$ overweight, and ≥30 obese[23]. Furthermore, due to the age-related changes in height, body composition, and curvature of the vertebral column in the elderly, we performed additional analyses for those elderly >75 years of age using an alternative BMI cutoff of ≥27 for the diagnosis of overweight and obesity[24,25]. To study central obesity, waist circumference was measured. Values >102 and 88 cm in men and women, respectively, were considered indicative of central obesity[8,26]. The relationship between the incidence of overweight or obesity, and socioeconomic and demographic factors, including gender, place of residence (urban or rural), literacy (literate or illiterate), employment status (employed or unemployed), type of living (alone, with family or any other form), use of supplements (vitamins, minerals, and omega 3 fatty acids) during the past 3 months, and source of income (Behzisty charity, Emdad Committee charity, retirement salary, family support or personal wealth), were investigated.

Data were analyzed using SPSS 13.0 for windows (SPSS Science, Apache Software Foundation, Chicago, IL). Data were presented as mean ± SD or number and percent (for categorical variables). Categorical variables were compared using the Chi-square test. Associations between different socioeconomic factors and the incidence of overweight or obesity were evaluated by binary logistic regression analysis using the forward conditional method. In all cases, $p < 0.05$ was considered to be statistically significant.

## RESULTS

The demographic data for the subjects, including their educational status and place of residence, are shown in Table 1. The mean age was 70.2 ± 7.8 years and mean BMI was 25.6 ± 7.6 kg/m$^2$.

**TABLE 1**
**Demographic and Anthropometric Values in Elderly Population of Razavi-Khorasan Province, Iran**

| | | Male | Female | Total |
|---|---|---|---|---|
| Age | | 71.4 ± 8.2 | 69.1 ± 7.3 | 70.2 ± 7.8 |
| BMI (whole population) | <19 | 127 (14.2) | 120 (11.9) | 247 (13.0) |
| | 19–24.9 | 464 (52.0) | 419 (41.4) | 883 (46.3) |
| | 25–29.9 | 237 (26.5) | 316 (31.2) | 553 (29.0) |
| | ≥30 | 65 (7.3) | 157 (15.5) | 222 (11.7) |
| BMI (≥75 years) | <19 | 127 (14.2) | 120 (11.8) | 247 (13.0) |
| | 19–26.9 | 496 (55.5) | 453 (44.8) | 949 (49.8) |
| | ≥27 | 270 (30.3) | 439 (43.4) | 709 (37.2) |
| Weight (kg) | | 63.1 ± 12.8 | 56.5 ± 13.4 | 59.6 ± 13.5 |
| Height (cm) | | 163.6 ± 7.4 | 150.8 ± 7.5 | 156.8 ± 9.8 |
| Waist circumference (cm) | | 90.0 ± 13.4 | 91.4 ± 14.5 | 90.8 ± 14.0 |
| Hip circumference (cm) | | 94.6 ± 10.5 | 95.8 ± 12.6 | 95.3 ± 11.7 |
| Literate | | 258 (28.3) | 135 (12.9) | 393 (20.1) |
| Urban | | 515 (56.2) | 604 (57.8) | 1119 (57) |

Values are expressed as mean ± SD or number (%).

The prevalence of overweight and obesity in our population sample were 28.9 and 11.7%, respectively. General (15.5 vs. 7.3%, $p < 0.001$) and central obesity (63.1 vs. 18.6%, $p < 0.001$) were more prevalent in women than men. Obesity was also more common among those who were literate compared with illiterate subjects (14.8 vs. 10.4%, $p = 0.014$).

In relation to the frequency of consumption of meals, the frequency of overweight or obesity was lower in those elderly consuming one meal per day (29.8%) compared with those who consumed two

(39.3%, $p = 0.04$) or three (42.5%, $p = 0.004$) meals per day. However, the difference in the incidence of overweight or obesity between those with two and three daily meals was not significant ($p = 0.23$).

### The Relationship between Obesity and Socioeconomic Factors

The relationship between the incidence of overweight or obesity with socioeconomic factors, including gender, place of residence, literacy, type of living, source of income, and use of supplements during the past 3 months, was assessed using forward conditional logistic regression analysis. Gender ($p < 0.001$), place of residence ($p < 0.001$), literacy ($p = 0.01$), and source of income ($p < 0.001$) were significantly associated with the incidence of overweight or obesity (Table 2). On the other hand, type of living ($p = 0.90$), employment status ($p = 0.59$), and use of supplements during the past 3 months ($p = 0.42$) were not found to be significantly related to the incidence of overweight or obesity. Results indicated that the incidence of overweight or obesity was more among females, urban dwellers, and literate subjects. In regard to the source of income, overweight or obesity was more prevalent among subjects with a retirement salary ($p < 0.001$). On the other hand, those subjects who received Emdad Committee charity had significantly lower incidence of overweight or obesity ($p = 0.002$). The same as general overweight and obesity, central obesity was also associated with gender ($p < 0.001$), place of residence ($p < 0.001$), literacy ($p = 0.006$), and source of income ($p < 0.001$). However no significant association was found for type of living ($p = 0.75$), use of supplements in the past 3 months ($p = 0.75$), and employment status ($p = 0.14$). As was the case for general overweight and obesity, the incidence of central obesity was more common among females, urban dwellers, and literate subjects. In regard to the source of income, central obesity was more prevalent among subjects with retirement salary ($p = 0.004$). On the other hand, those subjects who were receiving Emdad Committee charity had significantly lower incidence of overweight or obesity ($p < 0.001$).

In addition to the analyses mentioned above, we also performed an additional analysis for those elderly ≥75 years of age using an alternative BMI cutoff value of ≥27 for the diagnosis of overweight and obesity. The incidence of overweight and obesity was 17.8% among this group of elderly. Similar to the whole population, the combined prevalence of overweight and obesity was higher in females (19.5%) compared to males (16.5%), although this difference did not reach statistical significance ($p = 0.38$). In the logistic regression analysis, only literacy was significantly associated with the incidence of overweight or obesity ($p = 0.02$, Table 2), while the association with other socioeconomic factors, including gender ($p = 0.21$), place of residence ($p = 0.46$), type of living ($p = 0.66$), source of income ($p = 0.13$), use of supplements during the past 3 months ($p = 0.11$), and employment status ($p = 0.14$), did not reach statistical significance.

## DISCUSSION

The result of this study showed that the prevalence of overweight and obesity in the elderly people living in the Razavi-Khorasan province of northern Iran was 28.9 and 11.7%, respectively, with a higher occurrence in women than men. These findings are in accord with several other reports. In a study of Tunisian elderly, the prevalence of obesity was also reported to be higher in women (21.7%) than in men (9.4%)[27]. Similar results for the rate of obesity were also found in a Bahraini population (32 and 25% in women and men, respectively)[28]. In India, it has been shown that 34% of men and 40.3% of women are overweight or obese[9]. In the Spanish elderly, the prevalence of overweight and obesity in men was reported as 49 and 31.5%, respectively, and in women 39.8 and 40.8%, respectively[8], and the prevalence of central obesity was 48.4% in men and 78.4% in women[8]. In contrast, a European study of a Hungarian population showed that elderly men had a higher prevalence of obesity compared with women (18.1 and 15.4%, respectively)[29]. In regard to elderly Americans (60 years of age or older), both 1990 and 2000 statistics from the Third National Health and Nutrition Examination Survey

**TABLE 2**
**The Relationship between BMI and Some Socioeconomic Factors in the Elderly from Razavi-Khorasan Province, Iran**

| Variable | B | SE | Exp (B) | 95% CI for Exp (B) | *p* Value |
|---|---|---|---|---|---|
| **Whole population** | | | | | |
| Gender | | | | | |
| Female | 0.73 | 0.10 | 2.08 | 1.69–2.55 | <0.001 |
| Male* | | | | | |
| Place of residence | | | | | |
| Rural | –0.47 | 0.11 | 0.62 | 0.50–0.78 | <0.001 |
| Urban* | | | | | |
| Literacy | | | | | |
| Illiterate | –0.34 | 0.13 | 0.71 | 0.55–0.92 | 0.01 |
| Literate* | | | | | |
| Source of income | | | | | |
| Behzisty charity | –0.82 | 0.69 | 0.44 | 0.11–1.69 | 0.23 |
| Emdad committee charity | –0.53 | 0.17 | 0.59 | 0.42–0.82 | 0.002 |
| Retirement salary | 0.58 | 0.16 | 1.79 | 1.30–2.47 | <0.001 |
| Family support | –0.02 | 0.17 | 0.98 | 0.70–1.37 | 0.91 |
| Personal wealth | 0.25 | 0.16 | 1.28 | 0.94–1.75 | 0.11 |
| Other* | | | | | |
| **Elderly with the age ≥75 years** | | | | | |
| Literacy | | | | | |
| Illiterate | –0.76 | 0.31 | 0.47 | 0.25–0.87 | 0.02 |
| Literate* | | | | | |

* Considered as the reference variable. B: regression coefficient; SE: standard error; Exp (B): odds ratio.

(NHANES III) and NHANES 1999-Current (NHANES-C) indicate that obesity is more prevalent among females (25.3 and 33.4% in 1990 and 2000, respectively) compared with males (21.2 and 30.2% in 1990 and 2000, respectively)[10,30]. Furthermore, this increasing trend in the obesity rate is predicted to continue in the future[31]. For the U.K. elderly, statistics from a health survey in England (combined data for 1991 and 1992) imply that the prevalence of men and women (aged 65–74 years) with a BMI ≥25 kg/m$^2$ is 62 and 60%, respectively[11].

In this present study, overweight, obesity, and abdominal adiposity were associated with some socioeconomic variables, including gender, place of residence, literacy, and source of income. It appears that socioeconomic factors may affect the risk of overweight or obesity in different societies. In a recent Scandinavian study, it was determined that the prevalence of obesity was higher in the rural elderly[32]. The prevalence of obesity and abdominal obesity were also higher in rural Korean elderly compared to urban dwellers[33]. In Saudi Arabia, the prevalence of central obesity in the general population, based on waist circumference and the ratio of waist circumference to hip circumference, was found to be 34.4 and 43.5% respectively[34]. However, a recent study found no difference in the prevalence of obesity between urban and rural elderly of 10 European countries, which may be indicative of similar socioeconomics in both urban and rural areas of these countries[35]. The reason for the lower prevalence of obesity in the rural elderly in comparison with the urban-dwelling elderly in this present study is likely due to the traditional lifestyle of the rural dwellers in Razavi-Khorasan province. For example, they are more likely to be physically active and have less access to modern living facilities than the urban elderly.

The prevalence of overweight and obesity in this study was higher in the literate elderly than illiterate elderly, in accord with a study of the Bahraini population[28]. In contrast, the results from another recent study showed that low educational attainment is one of the predictive factors for obesity in the elderly[36]. The prevalence of obesity was also higher in the illiterate people compared with those who had received a university education among elderly Spanish subjects[8]. Moreover, in another study, the BMI and waist circumference in women had an inverse relationship with the level of education and social class[37]. Among Korean elderly, both general and abdominal obesity were inversely related to the level of educational attainment[15]. These differences for developed and developing countries may be explained by the possibility that educational attainment in developing countries is an indication of a better salary and more access to western-style food products, whereas educational attainment in developed countries may indicate a higher level of understanding of recent information on healthy nutrition, with individuals being more sensitive to food selection and physical activity.

The prevalence of overweight and obesity in the elderly who were living on their retirement salary was higher than those elderly with personal wealth, on family support, and elderly on charities support. The results are in accord with results from a recent study showing that the elderly on higher income were at risk of overweight and obesity[38]. Generally, in regard to the relationship between obesity and socioeconomic factors in the populations of the U.S. and U.K., in a large and ethnically diverse cohort of postmenopausal U.S. women (aged 50–79 years), it was reported that the prevalence of obesity varies markedly by ethnicity, educational level, and family income. In the same study, ethnicity, low educational level or family income, sedentary lifestyle, high parity, and a high percentage of calories from total and saturated fat were found to be independent predictors of obesity[16]. In another survey among U.S. adults, it was reported that low income and low education may be associated with both obesity and obesity comorbidities[17]. Furthermore, obesity prevalence was found to be higher in groups with lower socioeconomic status in the U.K., which is consistent with another previous study in which a strong inverse association was reported between socioeconomic status and central obesity[18,19].

This study had limitations. Selecting clusters from the whole area of the Razavi-Khorasan province was very difficult. Indeed, gathering data from rural areas with different languages made some questionnaires not very reliable, so they were removed from further analysis. In the present study, we measured height for those who were able to stand upright and not having kyphosis using a stadiometer, and used the demispan method for those who were not able to stand. Although there are reports about the overestimation of height by using demispan[39,40], in a recent study on 3346 subjects aged ≥65 years who participated in the Health Survey for England (HSE), Hirani and Mindell reported no significant differences in mean demispan equivalent height (DEH), and measured height neither in men nor in women. Moreover, they did not find any significant difference between BMIs calculated using measured height or DEH. Therefore, in a large nationally representative sample, they confirmed that demispan measurement may serve as a useful estimate of stature in subjects (particularly women) aged ≥65 for BMI calculations[41]. Another advantage of demispan measurement for the calculation of BMI in the elderly is less decline with age compared with height[42].

Finally, regarding the results obtained from this study, it appears necessary to develop preventative strategies to reduce adiposity within the elderly population. Overweight and obesity are growing rapidly even in developing countries, and determination of the predictive factors, including socioeconomic determinants, may be the first step for planning preventive measures.

## ACKNOWLEDGMENTS

We would like to thank the Mashhad University of Medical Sciences, Avicenna Research Institute, and Beheshti University of Medical Sciences for their help and financial support.
